# University-based physical education as a structured temporal and spatial opportunity for shaping health-oriented lifestyles

**DOI:** 10.3389/fpubh.2025.1597480

**Published:** 2025-05-21

**Authors:** Lu Zhang, Tao Zhong, Kui Dong

**Affiliations:** ^1^College of International Studies, National University of Defense Technology, Nanjing, China; ^2^College of Sport and Health, Henan Normal University, Xinxiang, Henan, China; ^3^Department of Sports, Kunming Medical University, Kunming, China

**Keywords:** public health, healthy lifestyle, physical activity, obesity, healthy diet

## Abstract

**Background:**

Regular physical activity is widely recognized for its health benefits, including improved cardiovascular function and reduced risk of chronic diseases. However, insufficient physical activity remains prevalent among university students, many of whom fail to meet recommended guidelines. University-based physical education (PE) programs play a critical role in fostering active lifestyles and promoting health-oriented behaviors among young adults. This study aimed to examine the association between participation in PE classes and the adoption of health-oriented behaviors among Chinese college students, particularly in the 3^rd^ and 4^th^ years, when participation in PE is voluntary.

**Methods:**

A cross-sectional study was conducted with 1,902 3^rd^- and 4^th^-year university students (mean age: 20.94 years; 59.2% female). The survey collected data on demographic (age and gender), socioeconomic status, body mass index (BMI), and some health-related behaviors such as dietary habits, smoking status, physical activity levels, and frequency of PE class attendance.

**Results:**

Results showed 44.23% of students were physically inactive, 8.1% were smokers, and 61.18% had an unhealthy diet. Male students had higher rates of smoking, obesity, and unhealthy diets compared to females. Participation in PE classes was associated with lower risks of obesity, unhealthy diets, and physical inactivity. Logistic regression analyses confirmed that PE attendance was a protective factor against these unhealthy behaviors, with significant gender-based differences observed in lifestyle patterns.

**Conclusion:**

This study highlights the importance of PE in promoting healthier lifestyles among university students and underscores the need for continued access to structured PE programs throughout the undergraduate years.

## 1 Introduction

Numerous studies have highlighted the wide-ranging health benefits of regular physical activity, including enhanced cardiovascular function, reduced risk of chronic diseases, and improved cognitive performance (1). Additionally, recent studies have demonstrated a strong association between physical activity and improved mood, reduced anxiety and depression, and increased social support (2). Promoting physical activity has thus become a key strategy in reducing the incidence of non-communicable diseases, such as ischemic heart disease, stroke, type 2 diabetes, osteoporosis, and various cancers (3).

Despite these well-documented benefits, insufficient physical activity remains a widespread global concern (4). Insufficient physical activity is prevalent across various population groups, including university students. In China, approximately 75% of university students fail to meet general guidelines for physical activity (5).

One promising opportunity to improve the physical activity of university students is through physical education (PE) class. As awareness grows about the adverse consequences of sedentary behavior, PE programs at universities are increasingly recognition as essential platforms for fostering healthy habits among young adults (6). These programs also influence on shaping students' perceptions of the importance of physical fitness and overall wellbeing (7).

Studies indicate that structured PE programs within academic institutions can significantly improve students' physical fitness (8), leading to improvements in cardiovascular fitness, muscular strength, flexibility, and body composition (9). For example, Cornish et al. reported positive associations between PE participation and higher levels of cardiorespiratory fitness and muscular strength (10).

University life represents a pivotal developmental phase, where students often encounter various challenges, marked by new social experiences, shifting learning environments, and greater autonomy. During this time, the behaviors of students are often shaped by their peers and immediate surroundings (11). Additionally, as most university students are transitioning from adolescence to adulthood, they are generally young and healthy, factors which may lead to neglecting their personal wellbeing (12). Consequently, some studies have shown that university students are particularly vulnerable to unhealthy behaviors such as insufficient physical activity, unhealthy eating habits, alcohol consumption, and smoking (13).

In China, undergraduate PE classes are compulsory during the 1^st^ and 2^nd^ years of study. However, in the 3^rd^ and 4^th^ year, participation becomes optional. This transition presents an opportunity to investigate how voluntary participation in physical education classes relates to students' adoption of health-oriented behaviors. Thus, this study aimed to examined the association between participation in physical education classes and some variables such as age, gender, and economic status with the adoption of health- promoting lifestyle patterns among Chinese university students.

## 2 Materials and methods

### 2.1 Study design and participants

This cross-sectional study was conducted between April 1 to May 30, 2024 in Wenzhou University, China.

The required sample size was calculated using the population proportion estimation formula:


$$\begin{array}{l} \frac{Z^{2}.p\left(1-p\right)}{d^{2}}=n \end{array}$$

where *Z* was taken as 1.96 for a 95% confidence level, *p* was taken as 0.5, and *d* was taken as 0.03 (margin of error). Accordingly, the minimum sample size was obtained as 1,067 individuals. Considering a 20% non-response rate, the final sample size was increased to 1,521 individuals (14).

Only, 3^rd^ and 4^th^-year undergraduate students were included in the study, as physical education class was not mandatory for these students. Thus, 3^rd^ and 4^th^-year students were recruited using a classroom-based sampling method. In this method, questionnaires were administered during scheduled class.

Participants with acute mental problems conditions or severe physical limitations were excluded. Additionally, students who attended university fewer than 3 days per week were excluded to ensure sufficient exposure to university life and PE classes. Data were collected through questionnaires distributed in university classrooms across all departments. To ensure data quality, all questionnaires were reviewed at the time of collection and incomplete or inconsistent items were removed.

The purpose, procedures, potential benefits, and possible risks of the study were thoroughly explained to all the participants by the interviewer prior to data collection. Then, all participants provided written informed consent prior to their inclusion in the study. As all participants were university students aged 18 or older, consent was obtained directly from them, without the involvement of their parents. This study was conducted in accordance with the Declaration of Helsinki, and approved by the medical research ethics committee of Wenzhou University (No. WZU-20230610-33).

### 2.2 Measurements

#### 2.2.1 Socio-demographic characteristics

Participants reported their gender (binary: female/male), age (continuous variable, in years), and financial status, assessed on a five-point Likert scale ranging from “very poor” (1) to “very good” (5). Due to low response rates in “very poor” and “very good,” participants grouped into three categories—“good” (very good/good), “acceptable,” and “poor” (very poor/poor). This classification has already been used in some studies (15).

#### 2.2.2 BMI

BMI was calculated using self-reported weight and height (kg/m^2^). Then, participants were classified into four categories based on the Health Industry Standards of China: underweight (< 18.5 kg/m^2^), normal weight (18.5–24 kg/m^2^), overweight (24–28 kg/m^2^), and obese (≥28.0 kg/m^2^). For the purpose of logistic regression, BMI was further recoded as a binary variable: obese (BMI ≥ 28 kg/m^2^) and “non-obese” (BMI < 28 kg/m^2^) (16).

#### 2.2.3 Smoking status

Participants were asked about their smoking habits using this question, “Do you smoke?” with the following response options: (1) No, I have never smoked regularly, (2) No, I have stopped smoking, (3) Yes, occasionally, and (4) Yes, daily. For statistical analysis, smoking status was further dichotomized as smokers (current smokers—occasional or daily) and non-smokers (ex-smokers or never smokers) (17, 18).

#### 2.2.4 Dietary habits

Dietary assessment focused on three indicators: daily consumption of fruits, vegetables, and use of vegetable oils for cooking. According to the latest version of the Chinese Dietary Guidelines (2022), the recommended amount for daily consumption of fruits and vegetables in adults was 200–350 g and 300–500 g, respectively (19). Also, and according to this Guidelines, the use of light and vegetable oils was another criterion of healthy dietary. Participants who met all three criteria (consumption of suitable fruits and vegetables according to the guidelines and consumption of vegetable oils for cooking), were considered as healthy diet. This model has already been used in Chinese studies (19, 20).

#### 2.2.5 Physical activity

The physical activity level was measured using a short version of the International Physical Activity Questionnaire (IPAQ). In this questionnaire, frequency (days per week) and duration (minutes per day) of walking, moderate-intensity, and vigorous-intensity activities are assessed (4). For each of these activities, there is a METS (METs are multiples of the resting metabolic rate). The METs of walking, moderate, and vigorous physical activity equal 3.3, 4, and 8, respectively (4). To get the MET of each physical activity, multiply its coefficient by the total minutes of that physical activity per week. The total MET of physical activity equals the sum of the total MET of all physical activities mentioned in the questionnaire. Also, according to the scores obtained in the questionnaire, participants who reported < 600 MET-minute/week were categorized as physically inactive, in accordance with IPAQ guidelines and WHO recommendations for minimum physical activity levels in adults (4). Reliability for the IPAQ short version in Chinese adults has been established (correlation coefficients above 0.70) (21).

#### 2.2.6 Participation in PE classes

Students were asked to report the number of sessions they attended in PE classes during a month of the current academic year. In Chinese universities, participation in PE classes is mandatory in the 1^st^ and 2^nd^ years, but becomes optional from the third year onwards. Since the sample of this study consisted of 3^rd^ and 4^th^-year students, participation in PE classes was voluntary and varied greatly between individuals. Factors such as personal motivation, academic pressure, time conflicts with other classes, and access to courses played a role in determining participation. This difference in participation provided a suitable context for examining the association between PE class attendance and health-related behaviors.

### 2.3 Statistical analyses

Quantitative data were presented as M ± SD and categorical variables were summarized using percentage and frequencies. Differences between female and male were tested by Pearson's chi-square test. Univariate and multivariate logistic regression models were used to assess the association of the number of sessions attended in PE classes, age, and gender (male/female) with smoking status (smoker/non-smoker), dietary habits (healthy/unhealthy), physical activity (active/inactive), and obesity (obese/non-obese). These recordings were done with the aim of simplifying analysis and comparison with existing literature, and not because of the existence of ceiling or floor effects in the data distribution. The results of logistic regression models are presented as odds ratios (OR) and 95% confidence intervals (95% CI). Statistical analysis was performed with the SPSS version 26.0.

## 3 Results

A total of 2,100 questionnaires were distributed, of which 1902 were valid and included in the analysis, yielding an effective rate of 90.57% (age = 20.94 ± 0.51 years; female: 59.2%). Table 1 presents the descriptive statistics of socio-demographic characteristics and health-related variables. Among the participants, 20.9% (*n* = 397) reported having poor financial condition. Regarding BMI, 10.3% (*n* = 196) were underweight, 65.6% (*n* = 1,248) had normal weight, 15.8% (*n* = 300) were overweight, and 8.3% (*n* = 158) were classified as obese. The prevalence of physical inactivity was 44.23% (*n* = 841), and the mean participation in PE class was 1.952 ± 1.258 days per month. The prevalence of smoking was 8.1%. Regarding dietary habits, the frequency of “daily” fruit, vegetable, and vegetable oil consumption was 58.3%, 41.9%, and 61.23%, respectively. The prevalence of unhealthy diet patterns (as defined in the Methods Section) was 61.18%. Regarding gender differences, the prevalence of smoking was 3.12% among females and 13.08% among males. Similarly, the prevalence of unhealthy dietary habits was lower among females (53.29%) compared to males (69.07%). In terms of BMI, the prevalence of obesity was 5.8% among females and 10.5% among males (Table 1).

**Table 1:** The descriptive statistics for socio-demographic characteristics and health-related information.

**Variable**		**All (*n =* 1,902)**	**Female (*n =* 1,126)**	**Male (*n =* 776)**	***P***
Age (year)		20.94 ± 0.51	21.03 ± 0.53	20.85 ± 0.5	0.219
Participate in physical education class (day/month)		1.952 ± 1.258	1.944 ± 1.249	1.96 ± 1.267	0.466
Financial condition % (n)	Good	16.2% (308)	18.1% (204)	14.3% (111)	0.339
	Acceptable	62.9% (1,196)	60.5% (681)	65.3% (507)	
	Poor	20.9% (398)	21.4% (241)	20.4% (158)	
BMI % (*n*)	Underweight	10.3% (196)	12.6% (240)	8.3% (158)	0.001^*^
	Normal weight	65.6% (1,248)	68.5% (1,303)	62.7% (1,192)	
	Overweight	15.8% (300)	13.1% (249)	18.5% (352)	
	Obese	8.3% (158)	5.8% (110)	10.5% (200)	
Physical activity % (*n*)	Active	55.77% (1,061)	56.19% (1,069)	55.35% (1,053)	0.616
	Inactive	44.23% (841)	43.81% (833)	44.65% (849)	
Smoking status % (*n*)	Non-smokers	91.9% (1,748)	96.88% (1,843)	86.92% (1,653)	0.001^*^
	Current smokers	8.1% (154)	3.12% (59)	13.08% (249)	
unhealthy diet % (*n*)	Yes	61.18% (1,164)	53.29% (1,014)	69.07% (1,314)	0.001^*^
	No	38.82% (738)	46.71% (888)	30.93% (588)	

^*^p < 0.05.

This study used logistic regression models to examine how some socio-demographic and participation in PE classes influence the likelihood of engaging in unhealthy behaviors such as smoking, unhealthy diet, physical inactivity, and obesity. This model was appropriate due to the binary nature of the outcome variables. Table 2 present the results of logistic regression models. Based on univariate analyses, the risk of smoking decreased by age expressed in years (OR = 0.95, 95% CI: 0.92–0.96).

**Table 2:** Binary logistic regression predicting obesity, unhealthy diet, and physical inactivity.

**Variables**	**Category**	**Smoking, OR (95% CI)**	**Obesity, OR (95% CI)**	**Unhealthy diet, OR (95% CI)**	**Physical inactivity, OR (95% CI)**
Age	Continuous (years)	0.95 (0.94,0.97)^***^	1.00 (0.91,1.01)	0.91 (0.96,0.98)	1.03 (1.21,1.23)
Gender	Male (vs. Female)	1.88 (1.11,1.31)^***^	2.95 (1.94,2.31)^***^	3.03 (1.79,2.59)^***^	0.88 (0.62,1.02)
Financial condition	Poor (vs. Good)	1.25 (0.91,1.17)	1.13 (0.82,1.31)	1.40 (1.16,1.32)	1.29 (0.97,1.43)
	Acceptable (vs. Good)	1.31 (0.83,1.38)	0.97 (0.59,1.12)	0.78 (0.53,1.19)	1.21 (1.66,1.81)
Physical education	Continuous (days)	1.59 (1.09,2.27)	3.81 (2.45,4.15)^***^	1.78 (1.12,2.92) ^***^	3.17 (2.12,4.24) ^***^

OR, odds ratio; CI, confidence interval; ^***^p < 0.001.

Also, male students had significantly higher odds of obesity (OR = 3.02, 95% CI: 2.19–3.22), unhealthy diet (OR = 2.19, 95% CI: 1.66–2.89), and smoking (OR = 2.01, 95% CI: 1.46–2.65), confirming strong gender-based disparities in health-related behaviors. Additionally, participation in PE classes was inversely associated with obesity (OR = 3.12, 95% CI: 2.48–4.66), unhealthy diet (OR = 1.53, 95% CI: 1.17–2.61), and physical inactivity (OR = 3.01, 95% CI: 1.69–3.47), suggesting a protective effect of consistent PE involvement. Interestingly, interaction effects between PE participation and gender were not statistically significant, indicating that the positive impact of PE holds across both sexes. Table 2 present the results of multivariate logistic regression models. Involving all variables, results were similar to the results of the univariate analyses.

## 4 Discussion

This study investigated the multifaceted effects of PE on university students' health- related behaviors. Despite existing challenges in program design, resource allocation, and student engagement, the findings underscore the significance of comprehensive PE programs as a core component of university curricula. The findings support the notion that PE plays a pivotal role in shaping students' activity patterns and broader lifestyle choices.

The present study revealed that participation in PE classes was associated with reduced risks of obesity, unhealthy dietary habits, and physical inactivity. To the best of our knowledge, this was the first study to evaluated the relationship between university PE participation and health-oriented behaviors among Chinese students. In addition to promoting physical health, PE programs can positively influence students' lifestyle choices, fostering habits that extend beyond the university years. Some studies have shown that sustained physical activity during university is associated with the maintenance of active lifestyles post-graduation (22), which is critical for preventing many chronic lifestyle-related diseases.

Well-designed PE can enhance physical fitness levels and encourage positive health choices, such as healthier eating habits, reduced sedentary time, and improved sleep duration (10). However, the success of such programs depends on several key elements, including the diversity of activities offered, personalization of training, and the integration of lifelong skills (23). Embedding behavior change theories into the curriculum in university further strengthens the sustainability of some healthy behaviors beyond the classroom setting (23).

Thus, the framing of physical education as a “temporally and spatially structured opportunity” is supported by the findings of this study. Physical education classes provide a specific time (in terms of time) and a specific place (in terms of space) for physical activity, which is especially important in the busy schedules of university life. Regular exposure to these structured environments can help establish routines, accountability, and long-term behavior change. Previous research also supports this view, showing that predictable and accessible opportunities for physical activity can lead to sustained participation and improved health outcomes (24).

The results of this study showed that physical inactivity was prevalent in 44.23% of students, which is higher compared to previous studies such as Li et al., where 38% of female Chinese university students were classified as physically inactive (25). While participant composition may explain part of the difference, other factors should also be considered. First, intense academic pressures in Chinese universities often lead to reduced time and motivation for physical activity among students (26). Second, the increasing use of digital technologies and screen time, especially during and after the COVID-19 pandemic, has been associated with an increase in sedentary lifestyles among young people (27). Finally, limited access to structured physical education classes and the lack of suitable sports spaces in some universities may also contribute to reduced physical activity levels (28). Thus, environmental, technological, and psychological factors all play a role in students' physical activity patterns. Therefore, tackling the phenomenon of inactivity is not possible by changing curricula alone, but also requires lifestyle interventions, improving infrastructure, and supporting mental health.

The prevalence of smoking in this study was 8.1%, with significantly higher rates among male students. This aligns with the findings of a national survey by China CDC study (2021) (29), which reported a smoking rate of among college students, with males demonstrating higher smoking (29). However, some studies reported higher rates; for example, Song et al. reported that 29.8% of college students smoked or used e-cigarettes (30). Differences in measurement protocols may account for this difference. The Cultural norms may also influence these gender differences (31). In many Chinese communities, female smoking is socially stigmatized, which may suppress both actual prevalence and self-reporting (30).

Regarding dietary patterns, 61.18% of university students were classified as having unhealthy dietary patterns. This result consistent with previous studies conducted in countries such as Ethiopia (32), Saudi Arabia (33), and United Kingdom (34), all of which reported low intake of fruits and vegetables among university students. In line with existing studies, male students in our sample were more likely to exhibit unhealthy dietary habits. This trend is in line with previous studies showing gender differences in food preferences, with women often opting for foods that are considered healthier (35). Females tend to make healthier food choices, possibly due to higher health awareness and societal expectations (36).

The prevalence of overweight and obesity in this study was 24.1%. Also, female students had lower obesity rate compared to male students, reflecting patterns observed in previous studies (37–39).

This gender difference in prevalence of overweight and obesity may be partly explained by sociocultural factors. The development of media has established a prevailing notion that being thinness is a fundamental criterion of beauty, particularly for women. This belief is held by many women who perceive slender bodies as more attractive. Furthermore, women with slender bodies are likely afforded greater opportunities in society, including increased access to employment opportunities that prioritize a slim shape. As a result, women may be more motivated to adopt weight-control behaviors, driven not only by health but also by societal and professional expectations (40).

Despite the strengths of this study, several limitations must be acknowledged. First, the cross-sectional design does not allow for inferences of causality. Second, the use of self-reported measures for key variables such as physical activity may introduce recall or social desirability bias. Third, despite the large sample size, the findings may not be generalizable to students in other regions or universities with different demographic characteristics. Additionally, some important confounding variables such as academic stress, family history of illness, and mental health status were not assessed and thus not adjusted for in the analysis. Finally, while key demographic factors were controlled for, the interaction effects between variables were not comprehensively investigated and should be explored in future research to gain a deeper understanding of these observed associations.

The results of this study showed that participation in physical education classes is significantly associated with reduced obesity, physical inactivity, and unhealthy diet among students. These findings highlight the key role of physical education in shaping health-oriented behaviors—especially in the third and fourth years, when attendance at these classes is optional. Accordingly, it is suggested that universities extend structured physical education programs to the upper years and offer them on an optional but recommended basis. These programs should focus on healthy lifestyle education, goal setting, and habit formation, and be aligned with the students' curriculum and academic pressure. Also, given the higher prevalence of obesity, smoking, and unhealthy diet among male students, providing targeted support for this group is essential. University-wide health campaigns, peer-led health programs, and providing safe and accessible sports spaces could be practical solutions to translate these findings into practical policies.

## Data Availability

The original contributions presented in the study are included in the article/Supplementary material, further inquiries can be directed to the corresponding author.
