# Supplementary material for: University-based physical education as a structured temporal and spatial opportunity for shaping health-oriented lifestyles
Source: Front Public Health. 2025 May 21;13:1597480. doi: 10.3389/fpubh.2025.1597480 (PMC12133991; doi:10.3389/fpubh.2025.1597480)
Supplement: Supplementary file 1 [file Table_1.docx]

# STROBE Checklist for Observational Studies

## Title and Abstract

Recommendation: Indicate the study’s design with a commonly used term in the title or abstract.

Manuscript Response: The study design ('cross-sectional') is clearly stated in both the title and abstract.

## Background/Rationale

Recommendation: Explain the scientific background and rationale for the investigation.

Manuscript Response: This item is addressed in the manuscript's corresponding section (see introduction).

## Objectives

Recommendation: State specific objectives, including any pre-specified hypotheses.

Manuscript Response: This item is addressed in the manuscript's corresponding section (introduction).

## Study Design

Recommendation: Present key elements of study design early in the paper.

Manuscript Response: This item is addressed in the manuscript's corresponding section (see Methods).

## Setting

Recommendation: Describe the setting, locations, and relevant dates.

Manuscript Response: This item is addressed in the manuscript's corresponding section (see Methods).

## Participants

Recommendation: Give eligibility criteria, and the sources and methods of selection.

Manuscript Response: This item is addressed in the manuscript's corresponding section (see Methods).

## Variables

Recommendation: Clearly define all outcomes, exposures, predictors, potential confounders, and effect modifiers.

Manuscript Response: This item is addressed in the manuscript's corresponding section (see Methods).

## Data Sources/Measurement

Recommendation: Give sources of data and details of methods of assessment.

Manuscript Response: This item is addressed in the manuscript's corresponding section (see Methods).

## Bias

Recommendation: Describe efforts to address potential sources of bias.

Manuscript Response: This item is addressed in the manuscript's corresponding section (see Methods).

## Study Size

Recommendation: Explain how the study size was arrived at.

Manuscript Response: This item is addressed in the manuscript's corresponding section (see Methods).

## Quantitative Variables

Recommendation: Explain how quantitative variables were handled in the analyses.

Manuscript Response: This item is addressed in the manuscript's corresponding section (see Methods).

## Statistical Methods

Recommendation: Describe all statistical methods, including those used to control for confounding.

Manuscript Response: This item is addressed in the manuscript's corresponding section (see Methods).

## Participants (Results)

Recommendation: Report numbers of individuals at each stage of study.

Manuscript Response: This item is addressed in the manuscript's corresponding section (see Results).

## Descriptive Data

Recommendation: Give characteristics of study participants.

Manuscript Response: This item is addressed in the manuscript's corresponding section (see Results).

## Outcome Data

Recommendation: Report numbers of outcome events or summary measures.

Manuscript Response: This item is addressed in the manuscript's corresponding section (see Results).

## Main Results

Recommendation: Give unadjusted estimates and, if applicable, confounder-adjusted estimates.

Manuscript Response: This item is addressed in the manuscript's corresponding section (see Results).

## Other Analyses

Recommendation: Report other analyses done – e.g., subgroup analyses, interactions, sensitivity analyses.

Manuscript Response: No formal sensitivity analysis was conducted, as all variables were categorized using standard guidelines (e.g., WHO and Chinese national references). This limitation is acknowledged in the Discussion section.

## Key Results

Recommendation: Summarize key results with reference to study objectives.

Manuscript Response: This item is addressed in the manuscript's corresponding section (see Results).

## Limitations

Recommendation: Discuss limitations of the study, taking into account sources of potential bias.

Manuscript Response: This item is addressed in the manuscript's corresponding section (see Discussion).

## Interpretation

Recommendation: Give a cautious overall interpretation of results.

Manuscript Response: This item is addressed in the manuscript's corresponding section (see Discussion).

## Generalisability

Recommendation: Discuss the generalisability of the study results.

Manuscript Response: This item is addressed in the manuscript's corresponding section (see Discussion).

## Funding

Recommendation: Give the source of funding and the role of the funders.

Manuscript Response: Funding sources are disclosed in the Acknowledgments section, and no funding body had a role in study design or interpretation.
